# Study on Fatigue Crack Propagation Behavior of Fiber/Al-Li Laminates Under Typical Overload

**DOI:** 10.3390/ma18163812

**Published:** 2025-08-14

**Authors:** Weiying Meng, Jiayi Tan, Sihui Li, Xiao Huang, Jiaying Wang

**Affiliations:** 1 School of Mechanical Engineering, Shenyang Jianzhu University, No. 25 Hunnan Middle Road, Hunnan District, Shenyang 110168, China; mengweiying025@163.com (W.M.); 19516928661@163.com (J.T.); w907217507@163.com (J.W.); 2 Key Laboratory of Fundamental Science for National Defense of Aeronautical Digital Manufacturing Process of Shenyang Aerospace University, Shenyang Aerospace University, No. 37 Daoyi South Street, Shenbei New District, Shenyang 110136, China; 3 School of Science, Shenyang Jianzhu University, No. 25 Hunnan Middle Road, Hunnan District, Shenyang 110168, China; 4 Aero Engine Corporation of China, No. 5 Landianchang South Road, Haidian District, Beijing 100097, China; huangx@aecc.cn

**Keywords:** Fiber/Al-Li laminates, single-peak tensile overload, single-peak compressive overload, fatigue crack propagation, prediction

## Abstract

Fiber metal laminates are applied in aerospace equipment due to their excellent crack propagation performance. However, during the service process of fiber metal laminates, the coupling between overload effect and fiber bridging effect makes the crack propagation behavior complex, which makes it difficult to predict. Addressing this issue, the fatigue crack propagation behavior of Fiber/Al-Li laminates under typical overload conditions was analyzed and predicted in this paper. Firstly, based on flight loading characteristics, fatigue crack propagation tests under constant amplitude and single-peak tensile/compressive overload were designed and conducted for Fiber/Al-Li laminates. The crack propagation behavior characteristics under typical overload conditions were analyzed and investigated. Secondly, the influence mechanism of thickness dimensions was revealed based on fatigue crack propagation characteristics under constant amplitude loading. A thickness size effect factor was introduced to improve the equivalent crack length model, where the crack propagation behavior of non-overload stages was simulated. Thirdly, improved Wheeler theory was adopted to characterize the overload hysteresis effect in the hysteresis zone under tensile overload; improved incremental plasticity theory was used to describe crack propagation behavior in the overload zone under compression overload. Finally, based on crack behavior characteristics under single-peak tensile and compressive overloads, the improved equivalent crack length model was combined to establish, respectively, the prediction models on crack propagation behavior under single-peak tensile and compressive overloads for Fiber/Al-Li laminates. Through experimental verification, the overall prediction error rate of the crack propagation model under tensile overload is up to 9.7%, and the overall prediction error rate of the crack growth model under compressive overload is up to 8.1%. Compared with similar models (not found) for thicker fiber metal laminates, the effectiveness and advancement of the proposed model are verified.

## 1. Introduction

Fiber metal laminates (FMLs) are composite materials made by alternately laying metal layers and prepreg layers in a predetermined order, which are prepared under specific pressure and temperature [1,2]. FMLs can achieve different material properties by changing the thickness, quantity and type of metal layers, the direction and system of fibers, and the thickness, number of layers and laying sequence of the prepreg layer. As an important aerospace material, the advantages of FMLs mainly lie in superior damage tolerance performance and impact damage performance [3], with fatigue damage tolerance performance being of greater concern for researchers.

**Figure 1:**
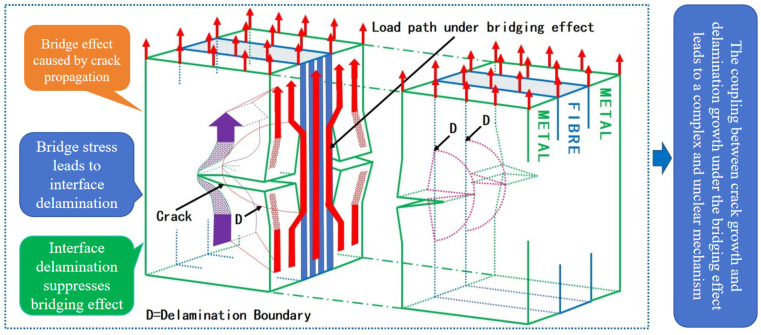
Schematic diagram of fatigue crack propagation mechanism in FMLs.

The excellent fatigue damage tolerance performance of FMLs is the result of fiber bridging [4,5]. As shown in Figure 1, two damage modes occurred in the fatigue process of the laminates: crack propagation in the metal layer and delamination propagation at the metal/fiber interface [6,7]. Fatigue crack propagation and interfacial delamination propagation interact and couple with each other under the bridging effect, which results in complex and unclear crack propagation mechanisms [8,9]. For aviation materials, overload loads are more commonly applied during service, which leads to damage interaction phenomena such as the overload hysteresis effect [10] and damage acceleration effect [11]. FMLs will be subjected to the interaction of the bridging effect and overload effect (hysteresis effect, acceleration effect) under overload load. The mechanisms of delamination propagation and crack propagation are more complex, which are difficult to explore. Therefore, it is difficult to build the model of crack propagation life reasonably.

Thorough research on the fatigue crack propagation performance of FMLs is an important means to reveal failure mechanisms and evaluate structural life. In previous studies, research on fatigue crack propagation performance mainly focused on the loading conditions of constant amplitude. Through combination with experimental testing and numerical simulation, influence mechanisms for different metal surface treatment methods on the interfacial strength and delamination behavior of fiber metal laminates are systematically studied by Liu Z et al. [12], and a traction–separation constitutive model is established that considers the correlation between metal surface roughness and mode I/II interlaminar properties. The mechanical properties of GLARE with different layup structures and prefabricated layered defects under shear buckling load was studied by Niazi M et al. [13], where the delamination propagation process was characterized by FM94 cohesive zone interface elements and a predictive model for laminate stability degradation based on layered radius and location was constructed. The fatigue delamination propagation rate of aluminum and carbon/glass fiber metal laminates was studied by Bieniaś and Dadej [14], and the flexibility, fatigue, and quasi-static interlaminar fracture of the laminates were determined by the end notch bending test. The fatigue crack propagation of GLARE under multiaxial fatigue conditions was studied by Kadhim MM et al. [15], where the crack propagation rate was characterized by using the effective stress intensity factor at the crack tip and the effective stress intensity factor values were calculated through the superposition of far-field stress intensity factor and the bridging stress intensity factor. Based on the crack propagation behavior characteristics under multiple-site damage (MSD), considering the influence of loading distribution from multiple crack on the bridging stress under single crack, the asymmetrical crack propagation behavior and the asymmetrical interface delamination propagation in the case of multiple cracks were predicted by Wang WD et al. [16]. Using the parameters n and C in the Paris formula was conducted by Yao LY et al. [17] as the crack propagation function; the quantitative expression of fiber bridging effect was realized by considering the characteristics of delamination state, the relationship between the Paris formula and the number of fiber bridges was revealed, and the mechanism between them was analyzed.

At present, research on the fatigue performance of FMLs under variable amplitude loading has emerged. Fatigue tests on GLARE 2/1 and GLARE 3/2 laminates under random load spectra were conducted by Cheng ZQ et al. [10] based on combining experiments with numerical simulations; linear and nonlinear fatigue models were introduced to analyze the crack propagation characteristics of the laminates and material progressive damage mechanism, and a hybrid modeling algorithm was proposed to effectively simulate the fatigue behavior and life under random load spectra for GLARE laminates. The fatigue performance relationship between FMLs and their metal materials under typical overload conditions was studied by Meng WY et al. [18], where the stress relationship between alloy and laminate materials was established based on classical laminate theory, and a phenomenological model on predicting the fatigue life of the laminates under corresponding overload conditions was constructed by considering the influence of bridging effect and laminate structure. Meanwhile, fatigue performance characteristics of fiber metal laminates under different loading modes (periodic single-peak tensile overload, periodic single-peak compression overload, and high–low overload) was analyzed by Meng WY et al. [19], and the damage accumulation criterion of laminate was modified based on the damage fracture coupling theory and the mechanism of the overload effect. Delamination propagation behavior and crack propagation behavior for unidirectional GLARE laminates were studied by Huang Y et al. under single-peak tensile overload [20]. The fatigue performance of composite–metal hybrid laminates under spectrum loading was researched by Seneviratne and Tomblin [21]. The Sendeckyj model was used to analyze the fatigue S-N curves under various stress ratios expected in the spectrum loading, and a mapping relationship between residual strength degradation and fatigue cycle times was established to achieve real-time tracking of residual strength degradation during loading service; finally a residual strength tracking model for hybrid laminates under variable amplitude fatigue loading was proposed.

These studies considered the effect of variable amplitude loading on the overall fatigue performance of FMLs from the overall level; however, research on the influence of specific loading on the crack propagation performance has not been focused on or in-depth studied for the laminates. In order to further apply and develop FMLs, it is necessary to analyze the effect of specific loading on crack propagation behavior and predict the crack propagation life under specific loading.

Firstly, the fatigue crack propagation behavior of Fiber/Al Li laminates under constant amplitude (CA) and typical overload was tested. Secondly, the characteristics of the crack propagation effect under typical overload conditions were analyzed for the laminates, and the effects of overload parameters (reference stress, overload ratio) on the fatigue crack propagation behavior of the laminates were studied. Then, the equivalent crack length model was improved by introducing the thickness size effect factor to characterize the crack propagation behavior of the laminates under constant amplitude. On this basis, the overload hysteresis effect of the crack hysteresis zone under tensile overload (TO) was characterized by improved Wheeler theory, and the crack propagation behavior in the compression overload (CO) zone was described by improving the incremental plasticity theory. Finally, prediction models of crack propagation behavior used in thicker FMLs were proposed, respectively, under single-peak tensile overload and compressive overload.

## 2. Fatigue Crack Propagation Test

### 2.1. Experimental Materials

**Figure 2:**
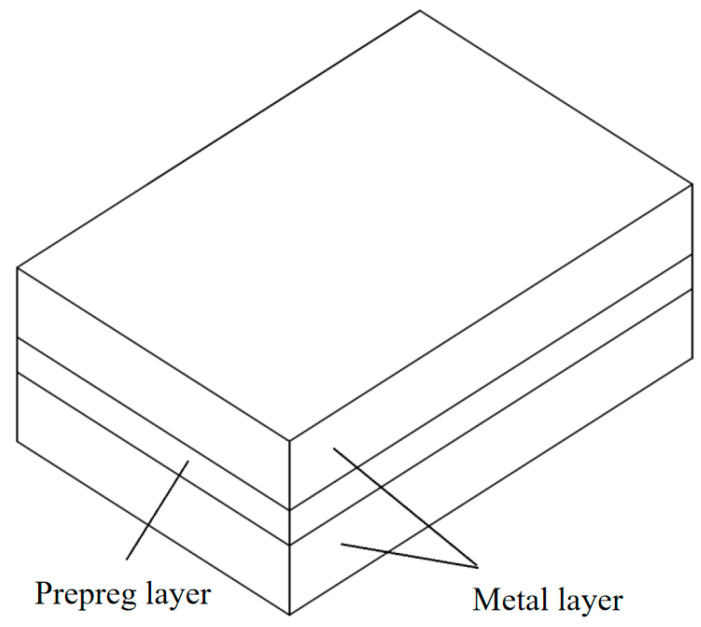
Schematic diagram of 2/1 laminate structure.

Fiber/Al-Li laminates with a 2/1 structure were used in the experiment. The laminate was composed of metal and fiber prepreg, where metal is two layers and fiber prepreg is one layer. The 2060-T8 Al-Li alloy was used as the metal layer, and the longitudinal direction of the material was used as the sampling direction of the Al-Li alloy plate. The SY-24/S4C9-1200 material was selected as the fiber prepreg layer, and the fiber direction of prepreg layer is 0°. The measured thickness of the 2060-T8 Al-Li alloy plate is 1.9 mm, and the chemical composition (mass fraction) of the 2060-T8 Al-Li alloy is shown in Table 1. The measured thickness of the fiber prepreg layer is 0.9 mm; S4 fiber and SY-24 adhesive are contained in the fiber prepreg layer. The fiber prepreg layer is prepared by the compression molding process; the properties of both materials are shown in Table 2. The performance test results of the Al-Li alloy and fiber prepreg layer are shown in Table 3, and the laminates with 2/1 structure are shown in Figure 2.

To ensure the excellent and stable mechanical properties for the material, the hot pressing molding process was adopted to prepare Fiber/Al-Li laminates used as the research object. Its manufacturing process mainly includes the following steps: surface treatment of the Al-Li alloy, pretreatment of prepreg, alternating placement of alloy layer and prepreg material layer, hot pressing consolidation (stress: 6 MPa, temperature: 120 °C, time: 30 min), cooling, and sampling. The adhesive used in the laminates is epoxy resin directly from the fiber prepreg layer. Finally, ultrasonic C-scan is used to check the bonding between the two layers to ensure that the material meets the requirements.

Different residual stresses were produced in each component material under high-temperature curing for FMLs, due to the different thermal expansion coefficient of each component material (the thermal expansion coefficient of the 2060-T8 Al Li alloy is 31.7 × 10^−6^/°C; the thermal expansion coefficient of fiber prepreg layer is 4.7 × 10^−6^/°C). The residual stresses of the metal layer are tensile stress. The residual stress in the metal layer of the Fiber/Al-Li laminates was measured using the Plustec instrument (Plustek, Taipei, China) with model number (μ-X360s), which is an X-ray residual stress measuring instrument. The testing principle is to use a two-dimensional detector to obtain a complete Debye ring. By comparing the differences between the Debye ring without stress and the deformed Debye ring under a stressed state, the changes in the crystal plane spacing under stress and the corresponding stress can be calculated. Through testing, we know that the residual stress value of the metal layer was 62.5 MPa, which will provide a data foundation for solving the effective stress ratio.

### 2.2. Specimen Form

**Figure 3:**
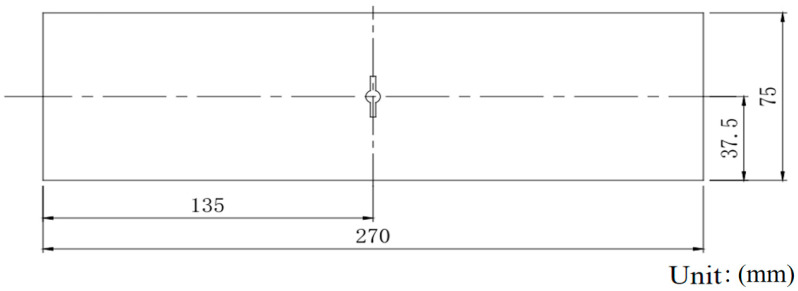
M(T) tensile specimen with central crack for laminates.

**Figure 4:**
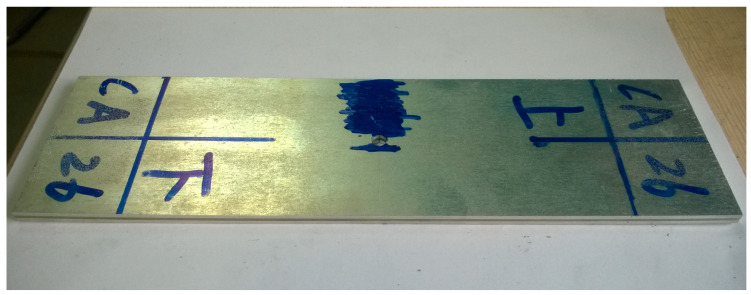
Practicality picture of crack propagation test specimen. Note (上 is up, 下 is down).

The M(T) tensile specimen with a center crack is used to the test the fatigue crack propagation for Fiber/Al-Li laminates. The specimen length is 270 mm, the specimen width is 75 mm, the diameter of the central round hole is 3 mm, and the saw-cut length is 2$a_{s}$ = 10 mm. The specimen form with a center crack is shown in Figure 3, and the crack propagation test specimen of the laminates is shown in Figure 4.

### 2.3. Experiment Equipment

**Figure 5:**
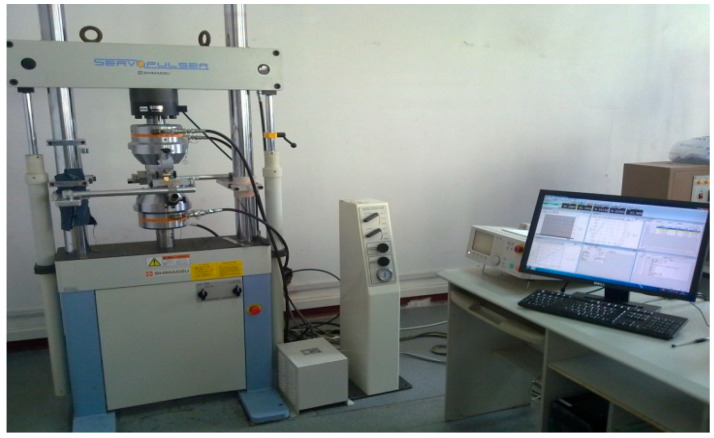
Low frequency fatigue testing machine in Shimadzu (Kyoto, Japan).

The test equipment of this study is the low frequency fatigue testing machine in Shimadzu. The low frequency fatigue testing machine adopts a control system with an electro-hydraulic servo, which can realize the test under constant amplitude loading and variable amplitude loading. The equipment parameters are as follows: the measurement range is ±100 kN, the dynamic error is <±3%, and the static error is <±0.5%. The test equipment is shown in Figure 5. In order to read the crack at the stable crack propagation stage and analyze the crack propagation increment, a JXD-B mobile microscope (Changchun Changcheng Education Instrument Co., Ltd., Changchun, China) with 20 times magnification is adopted, with a measurement accuracy of 0.01 mm. The measurement accuracy here has little effect on the results.

### 2.4. Experiment Scheme

**Figure 6:**
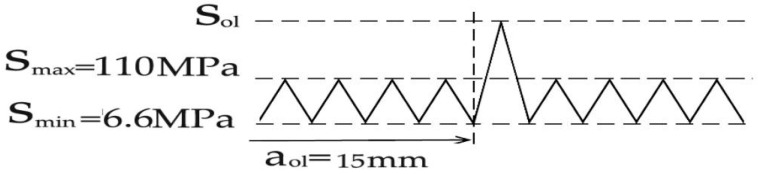
Schematic diagram of tensile overload loading.

**Figure 7:**
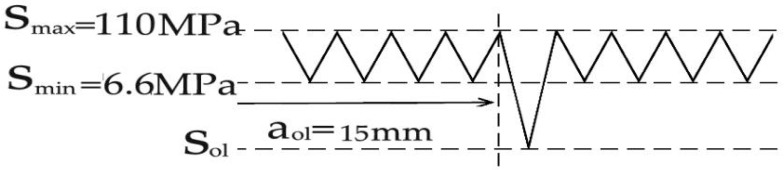
Schematic diagram of compression overload loading.

**Figure 8:**
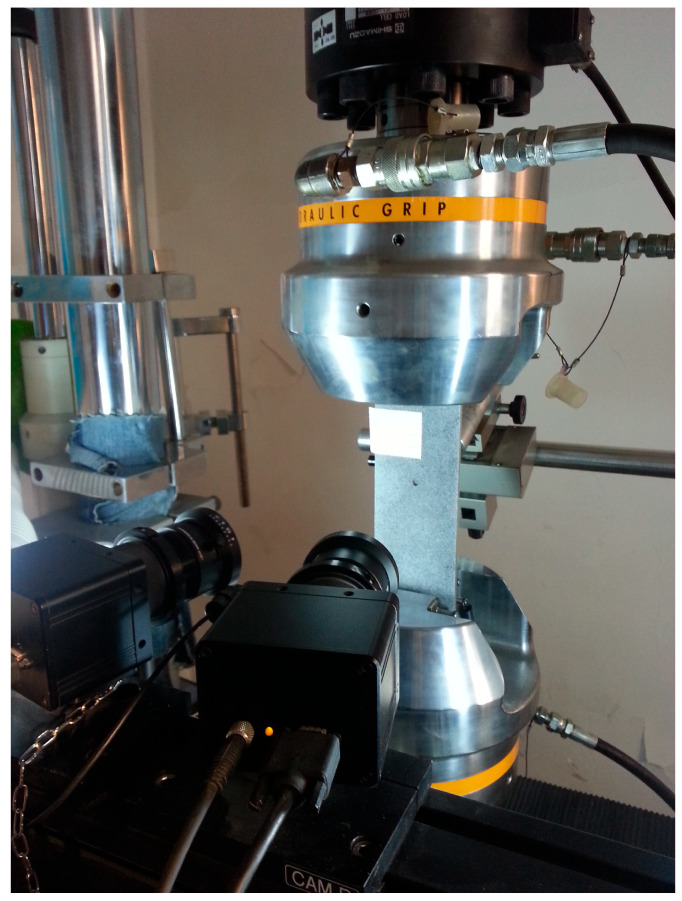
The testing process.

The test was conducted with reference to “Standard test method for fatigue crack propagation rates of metallic materials” (GB/T 6398-2017) [22]. The test is carried out at a room temperature and air environment under the loading with sine wave, and the test frequency is 10 Hz. Selection principles for loading frequency are as follows: sufficient loading response was ensured for the specimen, and the feedback loading of the specimen was consistent with the peak and valley values of the given loading. Constant loading at *R* = 0.06, single-peak tensile overload (*R*_ol_ = 1.4, 1.8), and single-peak compression overload (*R*_ol_ = −0.6, −1.8) were used for the loading mode in the test, where the overload position is *a*_ol_ = 15 mm. Taking the reference stress (110 MPa) and tensile overload ratio (1.8) as an example, the schematic diagram of tensile overload loading is shown in Figure 6. Taking the reference stress (110 MPa) and compression overload ratio (−1.8) as an example, the schematic diagram of compression overload loading is shown in Figure 7. When the crack grows to the specified crack length *a*_ol_ under constant loading, a corresponding loading *S*_ol_ = *R*_ol_ × *S*_max_ is applied according to the overload ratio *R*_ol_. After that, the constant amplitude crack propagation test is continued. The crack on the right side of the test specimen is taken as the observation object. The specific steps are as follows: (1) At the beginning of the experiment, an operation on a prefabricated crack is carried out, where the average length of the prefabricated crack is about 1.5–2 mm. During this period, two stresses greater than the test stress are applied in a gradually decreasing order. (2) After the prefabricated crack is completed, the applied stress is restored to the test stress. The testing machine is set to stop working every 5000 loading cycles. (3) Every time the machine stops, the crack length of the test specimen is read. The testing process is shown in Figure 8. Crack propagation data is read through the microscope behind the specimen in the figure.

### 2.5. Test Data Expression

During the experiment, the fatigue crack length *a* corresponding to different loading cycle times *N* can be read through a microscope. Due to the fact that the experimental data fitted by the secant method can more accurately reflect the changing trend of the experimental results, the secant method was used to represent the crack propagation rate d*a*/d*N* in this paper. The secant method can calculate the slope of a straight line connecting two adjacent experimental points through the *a*-*N* curve; the equation for the fatigue crack propagation rate is as follows:

(1) $$\frac{da}{dN}=\frac{\Delta a}{\Delta N}=\frac{a_{i+1}-a_{i}}{N_{i+1}-N_{i}}$$

where $a_{i}$ is the crack propagation length at $N_{i}$ cycles.

## 3. Analysis of Crack Propagation Performance Under Typical Overload

### 3.1. Crack Propagation Performance Under Single-Peak Tensile Overload

The corresponding fatigue crack length *a* and crack propagation rate d*a*/d*N* of Fiber/Al-Li laminates were drawn into *a*-d*a*/d*N* curves under constant amplitude loading and single-peak tensile overload at different reference stress levels (70 MPa, 110 MPa). The effects of single-peak tensile overload and different overload ratios *R*_ol_ on the constant amplitude fatigue crack propagation rate were analyzed by comparing the *a*-d*a*/d*N* data curves of different overload ratios (1.4, 1.8) at the same stress level (70 MPa, 110 MPa).

**Figure 9:**
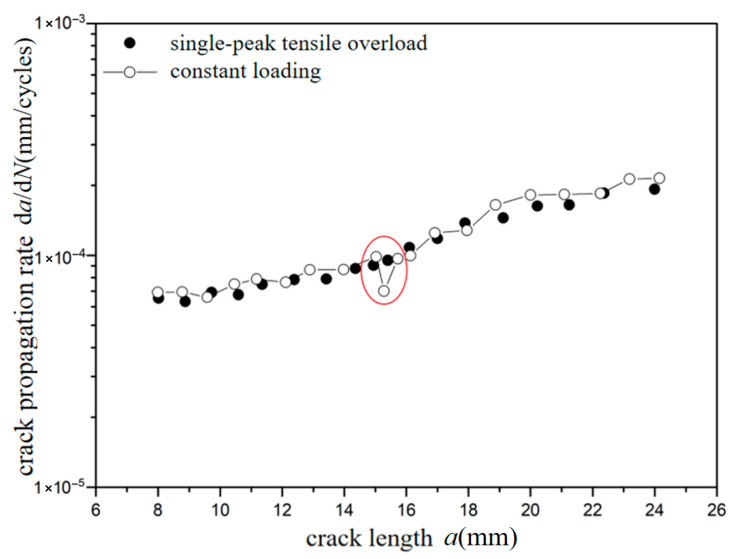
The rate of crack propagation (CA *S*_max_ = 70 MPa, TO *R*_ol_ = 1.4).

**Figure 10:**
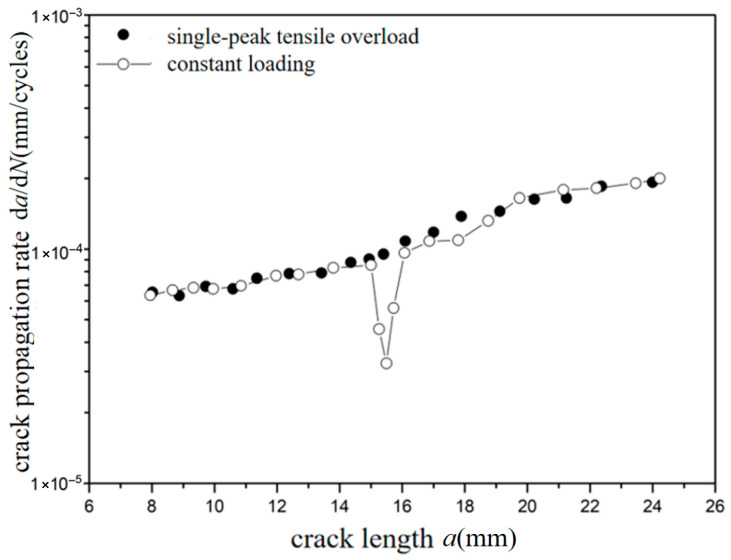
The rate of crack propagation (CA *S*_max_ = 70 MPa, TO *R*_ol_ = 1.8).

The *a*-d*a*/d*N* curves were compared under different overload ratios at reference stress 70 MPa for the laminates, as shown in Figure 9 and Figure 10. Under constant amplitude loading of 70 MPa, the fatigue crack propagation rate of the laminates has the tendency of slightly accelerating with the increase in crack length. When the crack length of the laminate is 15 mm, the overload hysteresis phenomenon is similar to that of the metal material after the single-peak tensile overload ratio *R*_ol_ = 1.4 is applied. From the perspective of fracture mechanics, when large tensile loading was subjected to the laminates, a large plastic zone at the crack tip of the metal layer occurs. This plastic zone causes a deceleration of the crack propagation rate, as shown in Figure 9. At the same crack propagation length, the laminate shows a greater overload hysteresis phenomenon similar to the metal material after the single-peak tensile overload ratio *R*_ol_ = 1.8 was applied. That is to say, when the laminate is subjected to a greater tensile loading, the tensile stress causes a larger plastic zone at the crack tip of the metal layer. This leads to a slower crack propagation rate and a longer influence time, as shown in Figure 10.

**Figure 11:**
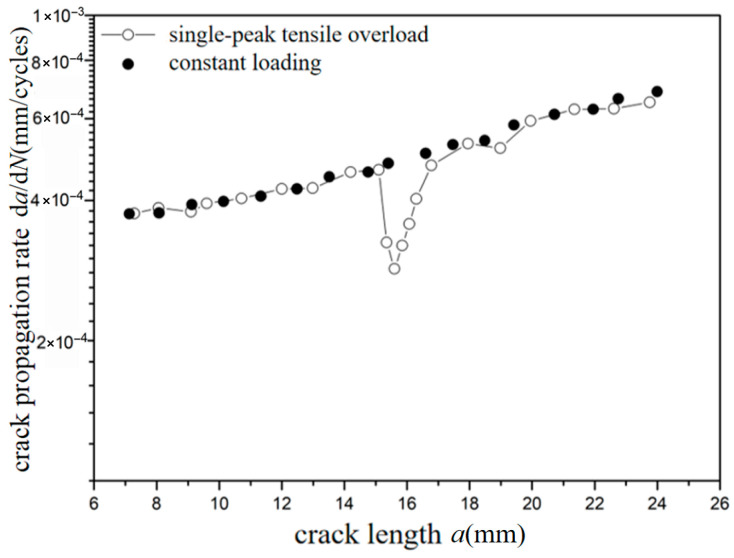
The rate of crack propagation (CA *S*_max_ = 110 MPa, TO *R*_ol_ = 1.4).

**Figure 12:**
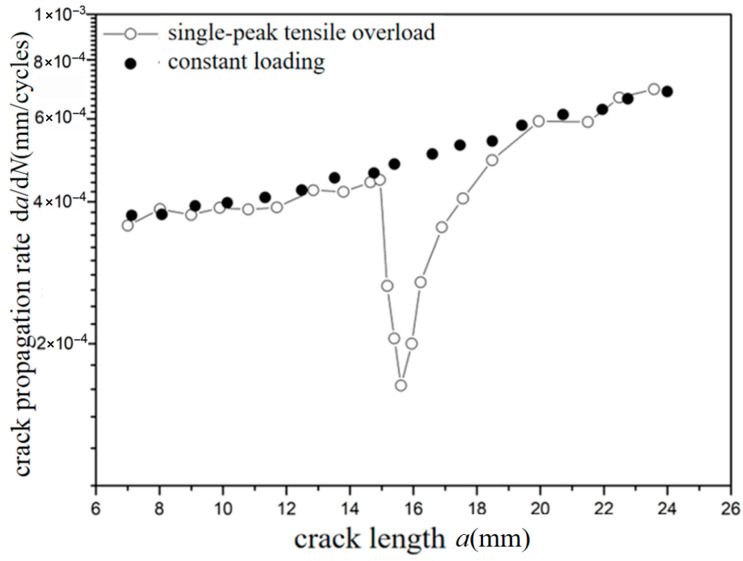
The rate of crack propagation (CA *S*_max_ = 110 MPa, TO *R*_ol_ = 1.8).

Similarly, the *a*-d*a*/d*N* curves were compared under different overload ratios at reference stress 110 MPa for the laminates, as shown in Figure 11 and Figure 12. The findings of the analysis show that, under constant amplitude loading of 110 MPa, the fatigue crack propagation rate for the laminates has the tendency of slightly accelerating with the increase in crack length. When the crack length of the laminate is 15 mm, crack propagation hysteresis behavior was observed more obviously than that of the laminate under the single-peak tensile overload ratio *R*_ol_ = 1.4 and constant amplitude stress with 70 MPa, as shown in Figure 11. This is because the increase in the reference stress causes the increase in the tensile loading under overloaded stress, which produces a larger plastic zone at the crack tip of the metal layer than 70 MPa. At the same crack propagation length, a greater overload hysteresis phenomenon was observed after the single-peak tensile overload ratio *R*_ol_ = 1.8 was applied, as shown in Figure 12. The reason is the same as in Figure 10.

In Figure 9, Figure 10, Figure 11 and Figure 12, the fatigue crack propagation behavior has an overload hysteresis effect after single-peak tensile overload loading is subjected to the effect. When the crack expands beyond the hysteresis zone, the crack propagation rate can be restored to that under constant amplitude loading. This phenomenon is caused by the overload plastic zone at the crack tip of the laminate, which leads to the fatigue propagation hysteresis effect after overload loading is applied. Simultaneously, the study reveals that crack propagation hysteresis effects occur more easily and become significantly enhanced with increasing reference stress; these hysteresis effects are markedly enhanced as the tensile overload ratio rises.

### 3.2. Crack Propagation Performance Under Single-Peak Compression Overload

The corresponding fatigue crack length *a* and crack propagation rate d*a*/d*N* of Fiber/Al-Li laminates under constant amplitude load and single-peak tensile overload at different reference stress levels (70 MPa, 110 MPa) were drawn into *a*-d*a*/d*N* curves. The effects of single-peak tensile overload and different overload ratios *R*_ol_ on the constant amplitude fatigue crack propagation rate were analyzed by comparing the *a*-d*a*/d*N* data curves of different overload ratios (1.4, 1.8) at the same stress level (70 MPa, 110 MPa).

**Figure 13:**
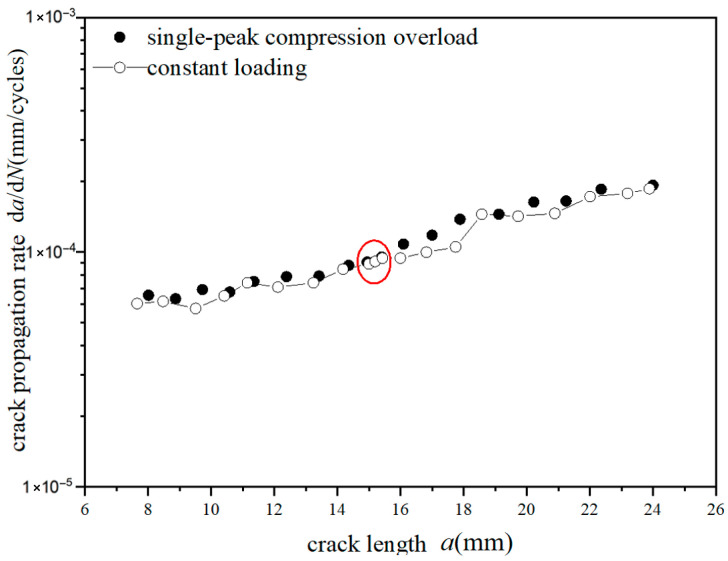
The rate of crack propagation (CA *S*_max_ = 70 MPa, CO *R*_ol_ = −0.6).

**Figure 14:**
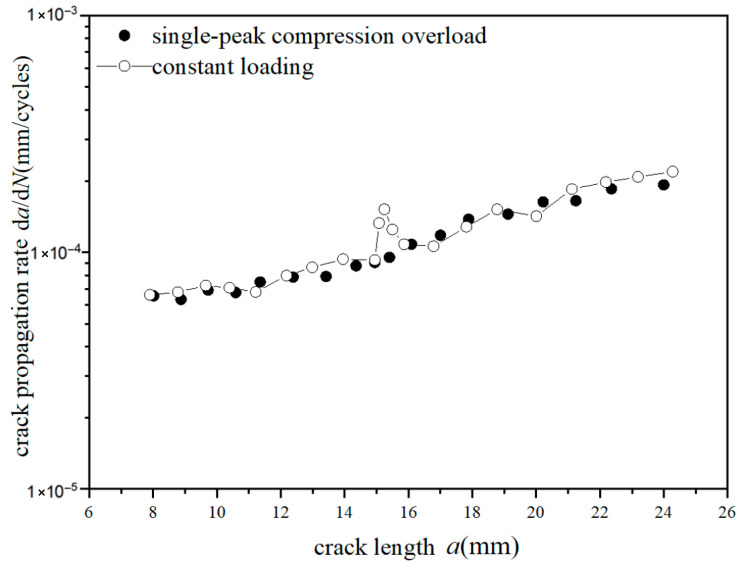
The rate of crack propagation (CA *S*_max_ = 70 MPa, CO *R*_ol_ = −1.8).

The *a*-d*a*/d*N* curves were compared under different overload ratios at reference stress 70 MPa for the laminates, as shown in Figure 13 and Figure 14. The crack propagation rates of constant amplitude loading, single-peak compression overload, and different overload ratios were analyzed. As shown in Figure 13, when the crack length of the laminate is 15 mm, the fatigue crack propagation rate did not show significant changes after the single-peak compression overload ratio *R*_ol_ = −0.6 was applied, which is similar to the crack propagation rate without overload under constant amplitude loading. From the perspective of fracture mechanics, when compressive overload loading was subjected to the laminates, the compressive stress is insufficient to cause plastic damage in the metal layer due to the counteracting effect of residual tensile stress in the metal layer. This makes no significant change for the fatigue crack propagation rate. As shown in Figure 14, at the same crack propagation length, the overload acceleration phenomenon similar to that of the metal material was observed after the single-peak compression overload ratio *R*_ol_ = −1.8 was applied. In other words, accelerated propagation occurred for crack propagation after the overload loading with the overload ratio of *R*_ol_ = −1.8 was applied, which makes the fatigue crack propagation rate increase rapidly. From the perspective of fracture mechanics, when the laminates were subjected to compressive overload loading, plastic damage was produced by compressive stress in the metal layer. This accelerates the fatigue crack propagation rate.

**Figure 15:**
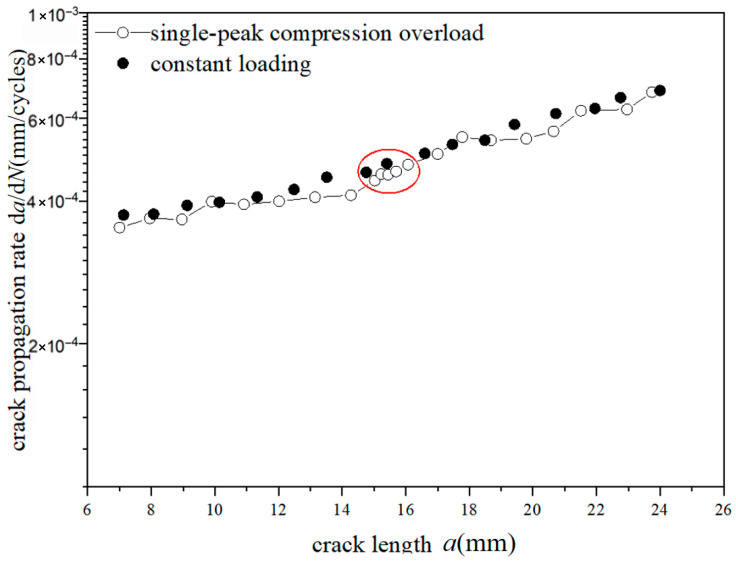
The rate of crack propagation (CA *S*_max_ = 110 MPa, CO *R*_ol_ = −0.6).

**Figure 16:**
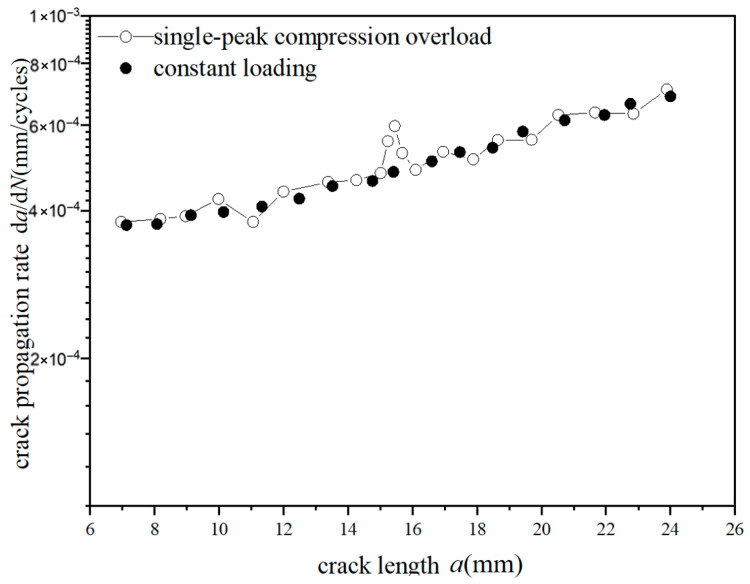
The rate of crack propagation (CA *S*_max_ = 110 MPa, CO *R*_ol_ = −1.8).

Similarly, the *a*-d*a*/d*N* curves were compared under different overload ratios at reference stress 110 MPa for the laminates, as shown in Figure 15 and Figure 16. As shown in Figure 15, when the crack length of the laminate is 15 mm, the fatigue crack propagation rate did not show significant changes after the single-peak compression overload ratio *R*_ol_ = −0.6 was applied, which is similar to the crack propagation rate without overload under constant amplitude loading. The reason is the same as in Figure 13. As shown in Figure 16, at the same crack propagation length, the overload acceleration phenomenon is obvious after the single-peak compression overload ratio *R*_ol_ = −1.8 is applied. When larger compressive overload loading is applied to the laminate, greater plastic damage is generated by compressive stress in the metal layer. This promotes further acceleration of fatigue crack propagation.

The crack propagation behavior characteristics at overload ratios of −0.6 and −1.8 under two different stresses of 70 MPa and 110 MPa were analyzed. It was found that there was no significant fluctuation for the crack propagation behavior at an overload ratio of −0.6. However, there was a significant fluctuation for the crack propagation behavior at overload ratio of −1.8, which means that crack propagation is accelerated. This phenomenon indicates the existence of a “certain value”. When the overload ratio is greater than this value, overload acceleration is generated. When the overload ratio is less than this value, the crack propagation behavior is not obviously changed. Similarly, when the crack extends to a certain length after overload, the crack propagation rate returns to that of the corresponding crack length under constant amplitude loading.

## 4. Fatigue Crack Propagation Model Under Overload

### 4.1. Equivalent Crack Length Model Considering Thickness Effect

For traditional thin FMLs, the fatigue crack will expand at an approximately constant rate [23]. Because the delamination propagation rate and fatigue crack propagation rate simultaneously depend on the bridging stress for the laminates, the balance between crack propagation and delamination propagation is reached by the fiber bridging effect under plane stress. The mutual adjustment of both makes the crack expand more stably.

From the equivalent crack length model considering interlayer performance, it can be seen that the effective stress intensity factor amplitude ∆*K*_eff_ of the laminate is constant when the crack stable propagates. The effective stress intensity factor equation of FMLs [23,24] is as follows:

(2) $$\Delta K_{\mathrm{eff}}=\frac{\sqrt{l_{0}}}{F\sqrt{(a-s)+l_{0}/F_{0}^{2}}}\Delta S\sqrt{\pi a}$$

where *a* is the fatigue crack length, *F* is the configuration factor of the laminate specimen, *s* is the length of sawing crack, *F*_0_ is the value of *F* when the fatigue crack length is the same as the sawing crack length, ∆*S* is the remote stress amplitude of the laminate, and *l*_0_ is the equivalent crack length of the laminate; the constant *l*_0_ can be obtained from the inverse calculation of the crack propagation rate d*a*/d*N* measured in the fatigue crack propagation test. *l*_0_ is expressed as follows:

(3) $$l_{0}=\frac{M^{2}}{1/F^{2}-M^{2}/F_{0}^{2}}(a-s)$$

where

(4) $$M=\Delta K_{\mathrm{eff}}/\Delta K=\frac{\sqrt[{n_{1}}]{da/dN}}{\sqrt[{n_{1}}]{C_{1}}{\left(1-R_{c}\right)}^{m_{1}-1}\left(F\Delta S\sqrt{\pi a}\right)}$$

(5) $$R_{c}=\frac{S_{\min}-S_{0}}{S_{\max}-S_{0}}$$

(6) $$S_{0}=-\frac{E_{\mathrm{la}}}{E_{\mathrm{Al}}}\sigma_{r,\mathrm{Al}}$$

where *C*_1_, *m*_1_ and *n*_1_ are the crack propagation constants for the component metals of the laminate, *R*_c_ is the effective cyclic stress ratio for the component metals of the laminate, *S*_0_ is the stress applied to the laminate when the actual stress of the component metals in FMLs is 0, *E*_la_ is the elastic modulus of the laminate, *E*_Al_ is the elastic modulus of the component metal, and *σ*_r,Al_ is the residual stress of the component metal; d*a*/d*N* can be obtained from the test.

However, when the thickness of the laminate increases, the plane stress problem transforms into the plane strain problem. This makes the stress intensity factor of remote stress increase, which leads to a small increase in the crack propagation rate of the laminates. Through the analysis of the test data, it is found that the fatigue crack propagation rate of the Fiber/Al-Li laminate under constant amplitude loading has a weak increasing tendency as the crack length increases. That is to say, the fiber bridging effect of the Fiber/Al-Li laminate is weak, which makes the delamination propagation and crack propagation of the laminate unable to reach a complete balance. This leads to the above phenomenon. Based on the characteristics of fatigue crack propagation behavior under constant amplitude for Fiber/Al-Li laminate, in order to make the equivalent crack length model suitable for the crack propagation characteristics of the laminate studied, the influence of the thickness effect on the crack propagation rate should be considered. Therefore, the thickness effect factor is introduced to improve the equivalent crack length model. Here we assume that the strength of the bridging effect is determined by the properties of the laminate itself.

For FMLs, when the interaction between delamination propagation and crack propagation leads to stable crack propagation, the expression of the fatigue crack propagation rate (d*a*/d*N*)_wen_ for FMLs under constant amplitude loading based on the Walker equation is as follows:

(7) $${\left(\frac{da}{dN}\right)}_{\mathrm{wen}}=C_{1}{\left[{\left(1-R_{c}\right)}^{m_{1}-1}\Delta K_{\mathrm{eff}}\right]}^{n_{1}}$$

where all parameters have been explained in the previous text.

The transformation of plane stress problems into plane strain problems disrupts the balance between delamination propagation and crack propagation, which leads to the crack propagation rate slightly accelerating. At the same time, the crack propagation rate increases linearly according to the analysis of crack propagation behavior. So, the expression of the fatigue crack propagation rate (d*a*/d*N*)_con_ of Fiber/Al-Li laminate under constant amplitude loading can be expressed as follows:

(8) $${\left(\frac{da}{dN}\right)}_{\mathrm{con}}=k{\left(\frac{da}{dN}\right)}_{\mathrm{wen}}$$

where *k* is the thickness effect factor. When the balance between delamination propagation and crack propagation is reached, *k* = 1. When the balance between delamination propagation and crack propagation cannot be reached, *k* is a variable. It relates to the crack length and stress level and is a performance parameter of the laminate. Research has found that the value of *k* is directly proportional to the fatigue crack length and inversely proportional to the stress level, which is a function of crack length and stress level. The expression for parameter *k* is

(9) $$k=\frac{110\left(Aa+B\right)t_{\mathrm{met}}}{S_{\max}}$$

where *S*_max_ is the peak value of remote stress applied by the laminate, and *t*_met_ is the thickness of the metal layer. Parameters *A* and *B* can be calculated from the crack propagation rate measured in the fatigue crack propagation test. Here, for the Fiber/Al-Li laminate, *A* = 0.0534, *B* = 0.5.

The balance between delamination propagation and crack propagation cannot be reached, which leads to a slight acceleration of crack propagation. The expression of the fatigue crack propagation rate d*a*/d*N* of Fiber/Al-Li laminate under constant amplitude loading is

(10) $${\left(\frac{da}{dN}\right)}_{\mathrm{con}}=\frac{110\left(Aa+B\right)}{S_{\max}}\left[C_{1}{\left[{\left(1-R_{c}\right)}^{m_{1}-1}\Delta K_{\mathrm{eff}}\right]}^{n_{1}}\right]$$

where all parameters have been explained in the previous text.

The crack propagation model proposed in this article under constant amplitude loading is based on the equivalent crack length model for fiber metal laminates. The prerequisite for the application of the proposed model is that fiber bridging occurs during the fatigue process, which is mainly used in case the laminate state changes from plane stress to plane strain due to size effects.

### 4.2. Crack Propagation Model Combined with Improved Wheeler Model Under Tensile Overload

For the fatigue crack propagation behavior of Fiber/Al-Li laminate under tensile overload loading, the overload hysteresis effect occurred under tensile overload. Therefore, on the basis of the improved equivalent crack length model mentioned above, the overload hysteresis model is used to introduce the effect of tensile overload on crack propagation under constant amplitude, so as to realize the prediction of the fatigue crack propagation rate of the laminates under tensile overload.

**Figure 17:**
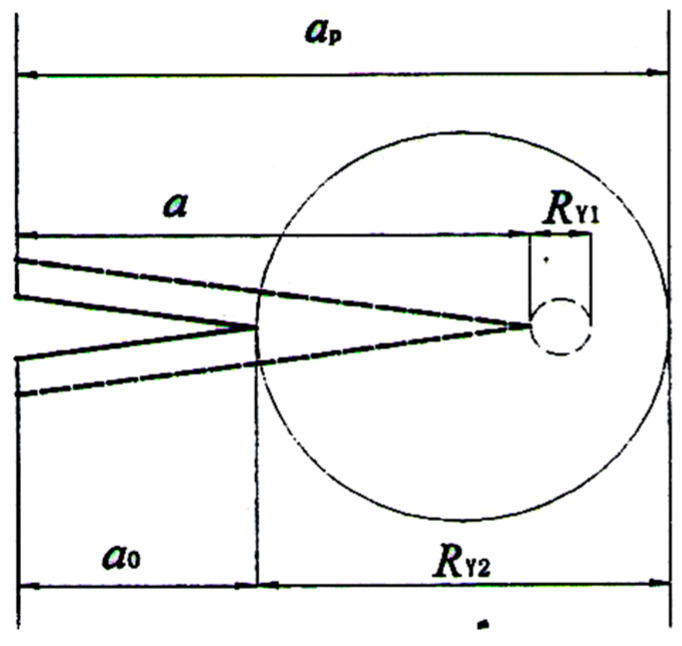
Schematic diagram of Wheeler model.

The overload hysteresis model is a simple interaction model. In this section, the Wheeler model is used as an overload hysteresis model. The Wheeler model assumes that [25] the following: (a) When the tensile overload is applied, a larger tensile plastic zone with diameter *R*_Y2_ is generated by tensile overload at the crack tip. When a smaller tensile plastic zone with diameter *R*_Y1_ generated by subsequent reference loading expands to the tangent of plastic zone with diameter *R*_Y2_, the crack propagation hysteresis disappears. (b) During the movement of the plastic zone with a diameter *R*_Y1_, the closer to the tangent of the larger plastic zone is, the weaker the crack propagation hysteresis effect will be. Its principle is shown in Figure 17.

The expression of the crack propagation rate (d*a*/d*N*)_ret_ in the crack propagation hysteresis period is

(11) $${\left(\frac{da}{dN}\right)}_{\mathrm{ret}}=C_{p}{\left(\frac{da}{dN}\right)}_{\mathrm{con}}$$

where ${\left(\frac{da}{dN}\right)}_{\mathrm{con}}$ is the crack propagation rate under constant amplitude loading. *C*_p_ is the crack propagation hysteresis coefficient, and its expression is as follows:

(12) $$C_{p}=\left\{\begin{array}{cc} {\left(\frac{R_{Y1}}{a_{p}-a}\right)}^{m}={\left(\frac{R_{Y1}}{a_{0}+R_{Y2}-a}\right)}^{m} & \;\;\;\;\;\;\;\;\;\;\;\;a+R_{Y1}<a_{p}\\ 1 & \;\;\;\;\;\;\;\;\;\;\;\;a+R_{Y1}\geq a_{p} \end{array}\right.$$

where *m* is the crack hysteresis index, *R*_Y2_ is the plastic zone generated by tensile overload, and *R*_Y1_ is the plastic zone generated by reference loading. According to the Irwin model, for the metal materials, the maximum plastic zone size at the crack tip is as follows [26]:

(13) $$\rho_{\max}=R_{Y2}=\frac{1}{\pi }\frac{K_{\max}^{2}}{\sigma_{s}^{2}}$$

where *K*_max_ is the stress intensity factor under the maximum loading; *σ*_s_ is the yield strength of metal materials. For FMLs, the crack propagation process is affected by bridging stress, so the concept of bridging stress is introduced to the Wheeler model. That is to say, the effective stress is used to characterize the maximum stress in the Irwin model. The effective stress is the sum of remote stress, residual stress, and bridging stress. The direction of bridging stress is opposite to the two. The proposed expression is as follows:

(14) $$\sigma_{\mathrm{effective}}=\sigma_{\mathrm{remote}}+\sigma_{r,\mathrm{Al}}+\sigma_{\mathrm{bridging}}$$

where $\sigma_{\mathrm{remote}}$ is the remote stress; $\sigma_{\mathrm{bridging}}$ is the bridging stress, which can be deduced in the literature [27]. Therefore, the stress intensity factor under the maximum loading is deduced as follows:

(15) $$K_{\max}=\left(\sigma_{\mathrm{remote}}+\sigma_{r,\mathrm{Al}}+\sigma_{\mathrm{bridging}}\right)\sqrt{\pi a}$$

where all parameters have been explained in the previous text. For FMLs, the size of the maximum plastic zone in the positive direction can be obtained by bringing Equation (15) into Equation (13), and the expression is

(16) $$\rho_{\max}=\frac{1}{\pi }\frac{{\left(\left(\sigma_{\mathrm{remote}}+\sigma_{r,\mathrm{Al}}+\sigma_{\mathrm{bridging}}\right)\sqrt{\pi a}\right)}^{2}}{\sigma_{s}^{2}}$$

where all parameters have been explained in the previous text.

**Figure 18:**
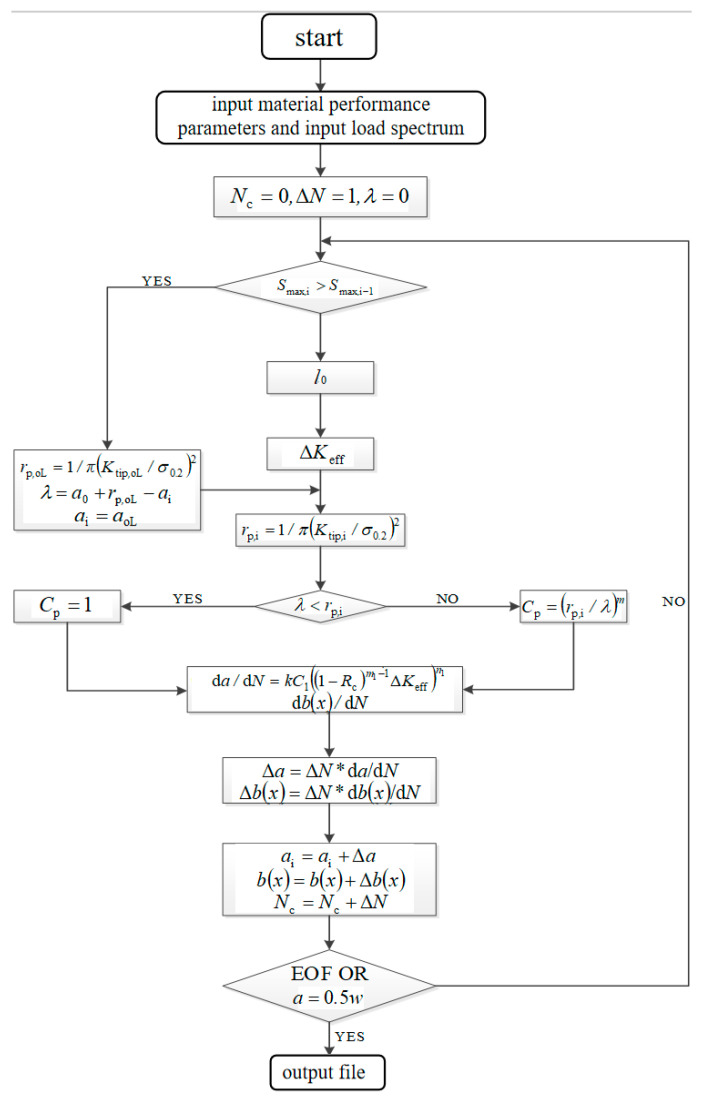
Flow chart of fatigue crack propagation prediction under tensile overload based on improved Wheeler model.

Fatigue crack propagation behavior under tensile overload is predicted based on an improved Wheeler model, and its implementation process is shown in Figure 18. When the model is subjected to a constant amplitude load, the crack propagation rate stabilizes, and an improved equivalent crack length model is used to predict its crack propagation rate. When the model recognizes a change in applied loading that is greater than the constant amplitude loading, a crack propagation model used in the overload hysteresis zone is adopted to predict the crack propagation rate in the hysteresis zone. When the crack extends to the edge of the hysteresis zone, the improved equivalent crack length model is used.

### 4.3. Crack Propagation Model Combined with Improved Incremental Plasticity Theory Under Compression Overload

For the fatigue crack propagation behavior of Fiber/Al-Li laminate under compression overload loading, the acceleration effect occurred under a certain reference stress at a large overload ratio. The overload hysteresis model cannot accurately predict the fatigue crack propagation behavior of the laminates. Therefore, on the basis of the improved equivalent crack length model mentioned above, the fatigue crack propagation behavior for the laminate is predicted by combing with the improved incremental plastic theory under compression overload in this section. For FMLs, the component metal for the laminates is still in a tensile state without external loading, which is caused by the existence of residual stress. Therefore, the modeling process of the prediction model on fatigue crack propagation under single-peak compression overload is as follows. Firstly, the effective cyclic stress ratio of the component metal for the laminate is determined under compression loading. Secondly, the relationship between the actual loading in the metal layer and the size of its plastic zone is given. Finally, the fatigue propagation behavior is predicted by combining the model with Paris theory.

(1) Effective cyclic stress ratio for metal layer under overload

When the residual stress of the metal layer is introduced, the effective cyclic stress ratio *R*_C_ for the laminate is shown in Equation (4) [28]. On this basis, the maximum compression loading of the effective remote stress for the component metal in the laminate is expressed as follows:

(17) $$\sigma_{\max,\mathrm{com},\mathrm{Al}}=\frac{E_{\mathrm{Al}}}{E_{\mathrm{la}}}\sigma_{\max,\mathrm{com}}+\sigma_{r,\mathrm{Al}}$$

where *σ*_max,com_ is the maximum compression loading. It can be seen from Equation (6) that for Fiber/Al-Li laminate, only when *σ*_max,com_ < −(*E*_la_/*E*_Al_)*σ*_r,Al_ under the single-peak compression overload loading, is the component metal layer of the laminate really in the compression state

(2) Correlation characterization between actual loading and plastic zone size

According to Irwin model, the maximum size of the reverse plastic zone at the crack tip in metal material is as follows [26]:

(18) $$\rho_{r}=\frac{1}{4\pi }\left(1-\gamma \frac{\sigma_{\max,\mathrm{com}}}{\sigma_{s}}\right)\frac{K_{\max}^{2}}{\sigma_{s}^{2}}$$

where *γ* is the material constant, which is related to the Bauschinger effect. For elastic/plastic materials, *γ* is taken as 1.8. Since the crack propagation process is affected by bridging stress, the concept of bridging stress is introduced to the incremental plasticity theory. That is to say, the effective stress is used to characterize the maximum stress in the Irwin model. The expression of effective stress is shown in Equation (14); the stress intensity factor under the maximum loading is shown in Equation (15). The maximum size of the reverse plastic zone can be obtained by bringing Equation (15) into Equation (18); the deduced expression is as follows:

(19) $$\rho_{r}=\frac{1}{4\pi }\left(1-\gamma \frac{\sigma_{\mathrm{effective}}}{\sigma_{s}}\right)\frac{{\left(\left(\sigma_{\mathrm{remote}}+\sigma_{r,\mathrm{Al}}+\sigma_{\mathrm{bridging}}\right)\sqrt{\pi a}\right)}^{2}}{\sigma_{s}^{2}}$$

where all parameters have been explained in the previous text.

(3) Prediction of crack propagation rate based on Paris theory

Crack propagation can be divided into two cases:

(1) When *S*_max_ ≥ *S*_0_ and σ_max,com_ > *S*_0_, i.e., *R* < 0, *R*_c_ ≥ 0, the minimum value of the effective stress intensity factor is greater than 0. According to the theory of equivalent crack length, the model of the fatigue crack propagation rate is still applicable for the laminates, and the expression is the same as the phenomenological model, which can be expressed as follows:

(20) $$\frac{da}{dN}=kC_{1}{\left(1-R_{c}\right)}^{m_{1}-1}\times {\left[\frac{\sqrt{l_{0}}}{\sqrt{\left(a-s\right)+l_{0}/F_{0}^{2}}}\left(S_{\max}-\sigma_{\max,\mathrm{com}}\right)\sqrt{\pi a}\right]}^{n_{1}}$$

where all parameters have been explained in the previous text.

(2) When *S*_max_ < *S*_0_ and *σ*_max,com_ < *S*_0_, i.e., *R*_c_ < 0. The fatigue crack does not propagate. A reverse plastic zone is created by compressive overload; the maximum size of reverse plastic zone can be obtained through Equation (19). When the reverse plastic zone is larger than the positive plastic zone, crack acceleration occurs. To describe this phenomenon, the influence factor of the reverse zone was introduced in the model.

The expression of the fatigue crack propagation rate (d*a*/d*N*)_acc_ for Fiber/Al-Li laminate under single-peak compression overload is

(21) $${\left(\frac{da}{dN}\right)}_{\mathrm{acc}}=C_{d}{\left(\frac{da}{dN}\right)}_{\mathrm{con}}$$

where *C*_d_ is the crack propagation acceleration coefficient, and its expression is

(22) $$C_{d}=\left\{\begin{array}{cc} {\left(\frac{R_{Y1}}{a_{0}\;+\;\rho_{r}\;-\;a}\right)}^{h} & \;\;\;\;\;\;\;\;\;\;a+R_{Y1}<a_{0}+\rho_{r}\\ 1 & \;\;\;\;\;\;\;\;\;\;a+R_{Y1}\geq a_{0}+\rho_{r} \end{array}\right.$$

where *h* is the crack acceleration index. Therefore, the prediction expression of fatigue crack propagation under single-peak compression overload for Fiber/Al-Li laminate is

(23) $${\left(\frac{da}{dN}\right)}_{\mathrm{acc}}=C_{d}kC_{1}{\left(1-R_{c}\right)}^{m_{1}-1}\times {\left[\frac{\sqrt{l_{0}}}{\sqrt{\left(a-s\right)+l_{0}/F_{0}^{2}}}\left(S_{\max}-\sigma_{\max,\mathrm{com}}\right)\sqrt{\pi a}\right]}^{n_{1}}$$

The implementation process of the fatigue crack propagation model under compression overload is as follows. When the model is subjected to a constant amplitude load, the crack propagation rate stabilizes, and an improved equivalent crack length model is used to predict its crack propagation rate. When the model recognizes a change in applied loading where the stress ratio corresponding to the applied loading is less than 0, the crack propagation model used in overload acceleration zone is adopted to predict the crack propagation rate in the acceleration zone. When the crack extends to the edge of the acceleration zone (reverse zone), the improved equivalent crack length model is used.

The crack propagation model used in the overload acceleration zone is based on improved incremental plastic theory. The prerequisite for the effectiveness of the model is that the compressive loading applied can still generate a reverse plastic zone at the crack tip after being offset by residual tensile stress.

## 5. Model Validation

To verify the accuracy of the proposed model, the crack propagation rate of Fiber/Al Li laminates was predicted under different overload modes and overload ratios, at reference stresses *S*_max_ = 70 MPa and *S*_max_ = 110 MPa, respectively.

### 5.1. Verification of Crack Propagation Model Under Single-Peak Tensile Overload

**Figure 19:**
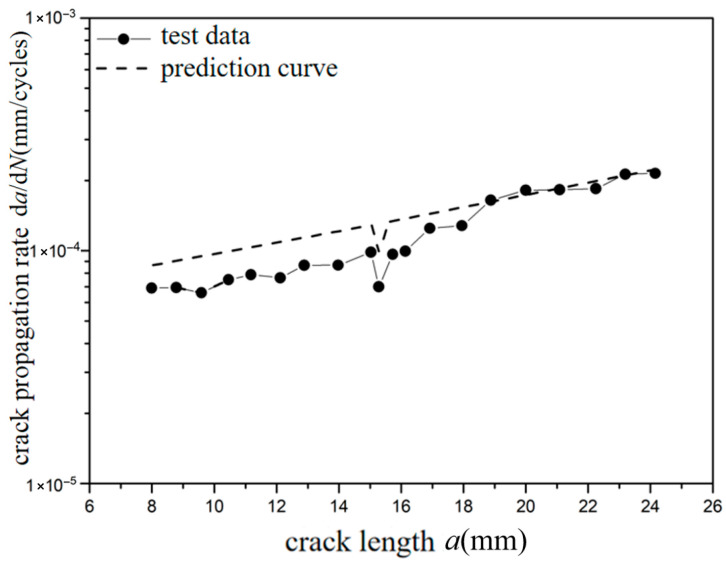
Prediction results of crack propagation rate (CA *S*_max_ = 70 MPa, TO *R*_ol_ = 1.4).

**Figure 20:**
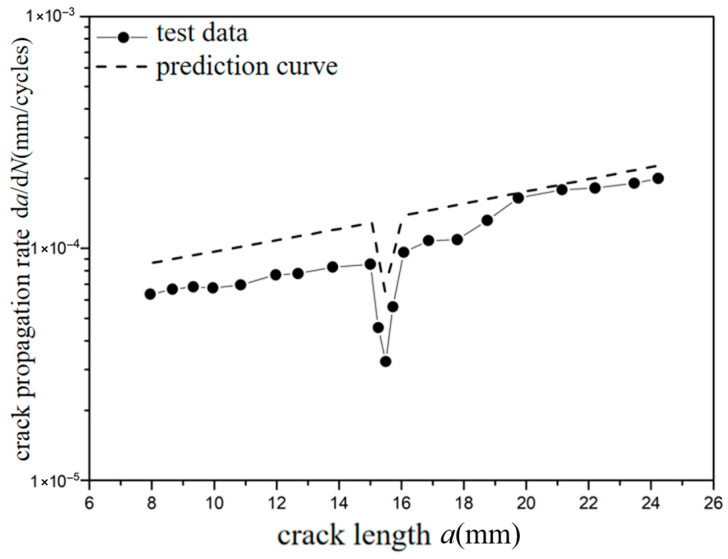
Prediction results of crack propagation rate (CA *S*_max_ = 70 MPa, TO *R*_ol_ = 1.8).

**Figure 21:**
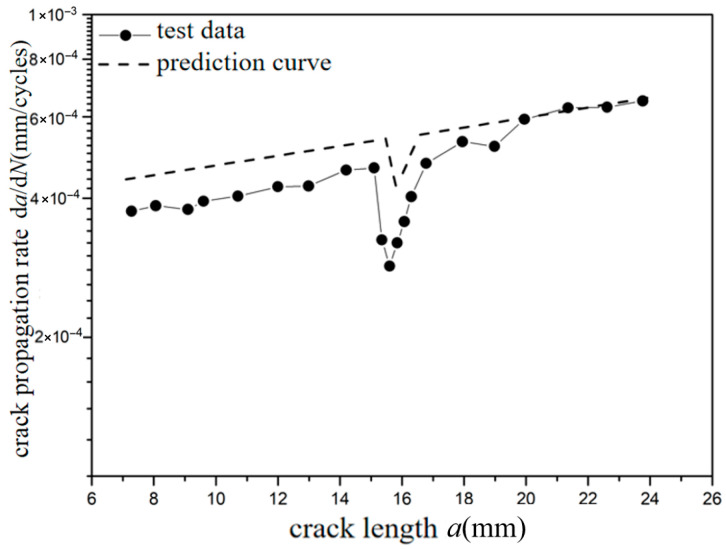
Prediction results of crack propagation rate (CA *S*_max_ = 110 MPa, TO *R*_ol_ = 1.4).

**Figure 22:**
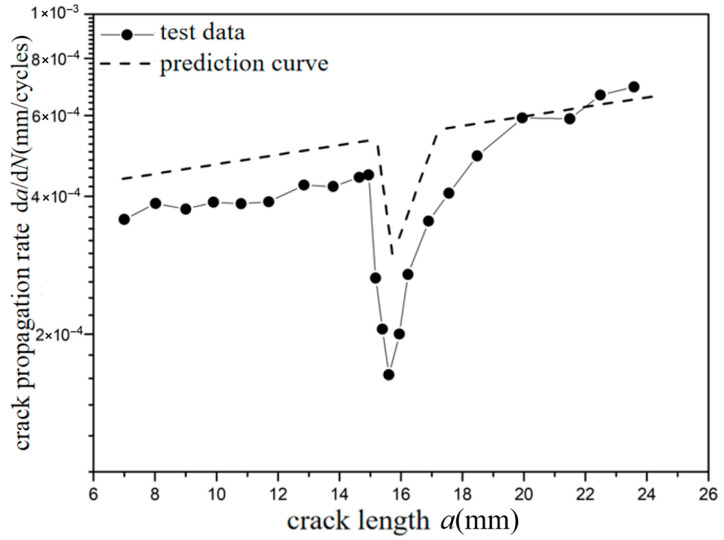
Prediction results of crack propagation rate (CA *S*_max_ = 110 MPa, TO *R*_ol_ = 1.8).

The test curves and predicted curves of *a*-d*a*/d*N* for Fiber/Al-Li laminates under reference stress (70 MPa, 110 MPa) with single-peak tensile overload ratios (1.4, 1.8) are, respectively, plotted in Figure 19, Figure 20, Figure 21 and Figure 22. It can be seen from the figure that under the lower reference stress 70 MPa and the higher reference stress 110 MPa, the prediction curves are in good agreement with the *a*-d*a*/d*N* test curves at the single-peak tensile overload ratio (1.4, 1.8). In Figure 19 and Figure 20, at the early stage of crack propagation (without overload stress), the average error rate between the prediction results and the two groups of test data is 18.3%; at the later stage of crack propagation (at the stage of stable propagation after overload applied), the average error rate between the prediction results and the two groups of test data is 4.9%. In Figure 20, at the overload stage, the average error rate between the prediction results and the test data is 11.8%. In Figure 21 and Figure 22, at the early stage of crack propagation, the average error rate between the prediction results and the two groups of test data is 14.6%; at the later stage of crack propagation, the average error rate between the prediction results and the two groups of test data is 4.3%. In Figure 22, at the overload stage, the average error rate between the prediction results and the test data is 10.2%. For the *a*-d*a*/d*N* curve with an overload ratio of 1.4, the tensile overload hysteresis effect occurred. The prediction curve is in good agreement with the test curve, and the prediction accuracy gradually improves as the crack propagates. For the *a*-d*a*/d*N* curve with an overload ratio of 1.8, the tensile overload hysteresis effect more obviously occurred; the predicted curves show a relatively large error compared with the test curve before the overload; the predicted curves have good agreement with the tensile overload trend of the test curve when the crack propagates in the plastic zone generated by tensile overload; there is a relatively small error between the predicted curve and the test curve after the crack propagates out of the plastic zone generated by tensile overload. Therefore, the overall average error rate of the prediction curve shall not exceed 15% (70 MPa, 11.6%; 70 MPa TO, 11.7%; 110 MPa,9.4%; 110 MPa TO, 9.7%); the proposed method is effective and advanced by considering the dispersion of the data and the consistency of the prediction trend.

The reasons for the differences between the prediction results and the test data are as follows: at the stage of crack propagation under constant amplitude loading, the crack propagation model under constant amplitude loading is improved by introducing the influence of thickness size based on the equivalent crack length model. So the accuracy of the model is mainly dependent on the data dispersion situation. In the early stage of crack propagation (without tensile overload stress), the average standard deviation of crack propagation data is relatively large, which leads to a high error rate for the prediction results. At the later stage of crack propagation (the stage of stable propagation after tensile overload stress applied), the average standard deviation is relatively small, which reduces the error rate of the prediction model. At the stage of the tensile overload effect, an improved Wheeler model is used to describe quantitatively the change in the plastic zone size caused by tensile overload. In summary, as the overload ratio and reference stress increase, the increase in plastic zone size is also reflected in reality. Therefore, the prediction results of the model can reasonably describe the hysteresis effect of crack propagation.

### 5.2. Verification of Crack Propagation Model Under Single-Peak Compression Overload

**Figure 23:**
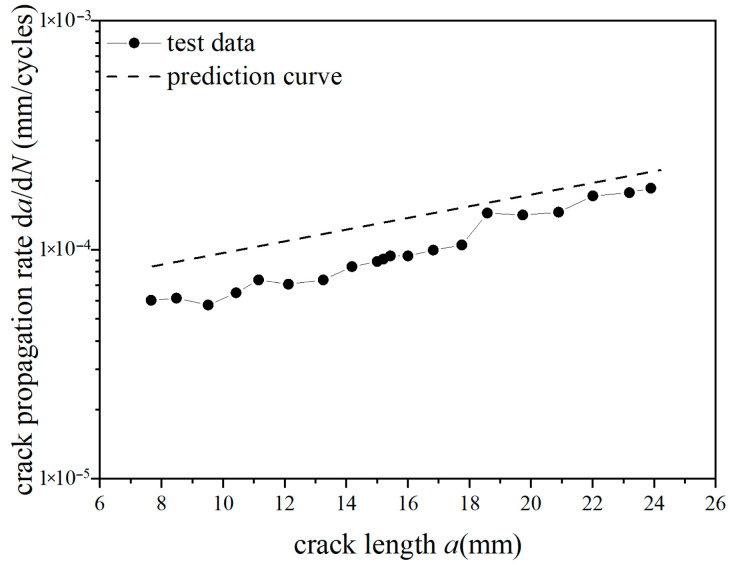
Prediction results of crack propagation rate (CA *S*_max_ = 70 MPa, CO *R*_ol_ = −0.6).

**Figure 24:**
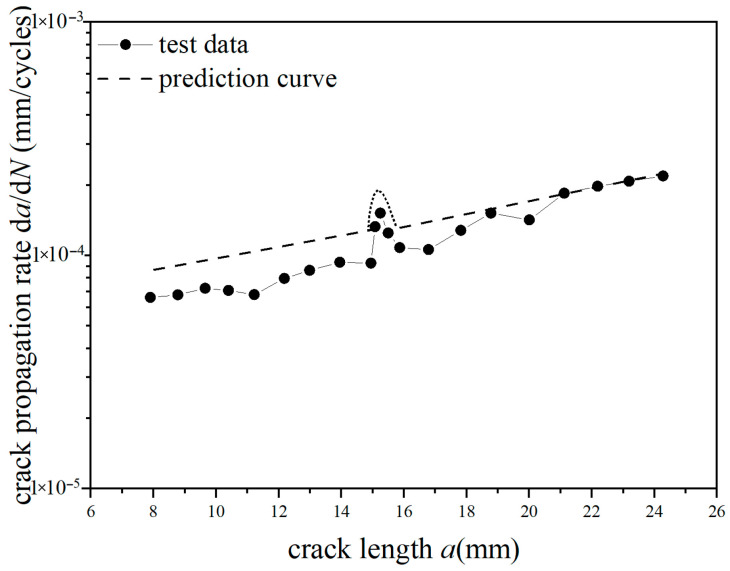
Prediction results of crack propagation rate (CA *S*_max_ = 70 MPa, CO *R*_ol_ = −1.8).

**Figure 25:**
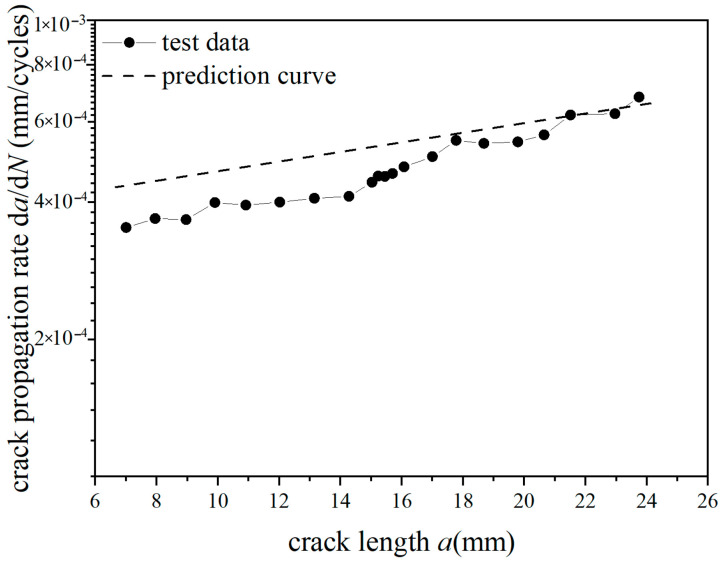
Prediction results of crack propagation rate (CA *S*_max_ = 110 MPa, CO *R*_ol_ = −0.6).

**Figure 26:**
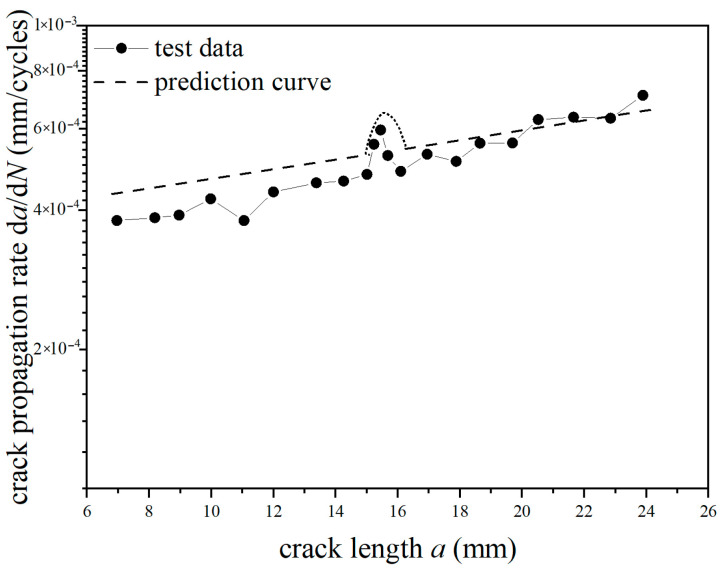
Prediction results of crack propagation rate (CA *S*_max_ = 110 MPa, CO *R*_ol_ = −1.8).

The test curves and predicted curves of *a*-d*a*/d*N* for Fiber/Al-Li laminates under reference stress (70 MPa, 110 MPa) with single-peak compression overload ratios (−0.6, −1.8) are, respectively, plotted in Figure 23, Figure 24, Figure 25 and Figure 26. It can be seen from the figure that under the lower reference stress 70 MPa and the higher reference stress 110 MPa, the prediction curves are in good agreement with the *a*-d*a*/d*N* test curves at the single-peak compression overload ratio (−0.6, −1.8). In Figure 23 and Figure 24, at the early stage of crack propagation, the average error rate between the prediction results and the two groups of test data is 19.2%; at the later stage of crack propagation, the average error rate between the prediction results and the two groups of test data is 5.6%. In Figure 24, at the overload stage, the average error rate between the prediction results and the test data is 10.5%. In Figure 25 and Figure 26, at the early stage of crack propagation, the average error rate between the prediction results and the two groups of test data is 13.8%; at the later stage of crack propagation, the average error rate between the prediction results and the two groups of test data is 2.6%. In Figure 26, at the overload stage, the average error rate between the prediction results and the test data is 7.8%. For the *a*-d*a*/d*N* curve with an overload ratio of −0.6, the compression overload acceleration effect did not occur. The prediction curve is in good agreement with the test curve, and the prediction accuracy gradually improves as the crack propagates. For the *a*-d*a*/d*N* curve with an overload ratio of −1.8, the overload acceleration effect more obviously occurred; the predicted curves show a relatively large error compared with the test curve before the overload; the predicted curves have good agreement with the compression overload trend of the test curve when the crack propagates in the plastic zone generated by compression overload; there is a relatively small error between the predicted curve and the test curve after the crack propagates out of the plastic zone generated by compression overload. Therefore, the overall average error rate of the prediction curve shall not exceed 15% (70 MPa, 12.4%; 70 MPa CO, 11.8%; 110 MPa, 8.2%; 110 MPa CO, 8.1%); the proposed method is effective and advanced by considering the dispersion of data and the consistency of the prediction trend.

The reasons for the differences between the prediction results and the test data are as follows: at the stage of crack propagation under constant amplitude loading, similarly, the accuracy of the crack propagation model under constant amplitude loading mainly depends on the data dispersion situation. In the early stage of crack propagation (without compression overload stress), the average standard deviation of crack propagation data is relatively large, which leads similarly to a high error rate for the prediction results. At the later stage of crack propagation (the stage of stable propagation after compression overload stress is applied), the average standard deviation is relatively small, which reduces the error rate of the prediction model. At the stage of the compression overload effect, the compression loading is offset by the residual stress of the laminate at an overload ratio of −0.6, which shows that the prediction result of the model does not cause the acceleration phenomenon. This is consistent with the overload trend of the test data. At an overload ratio of −1.8, the improved incremental plastic theory is used to describe quantitatively the change in the plastic zone size caused by compression overload, and to accurately define the relationship between crack propagation increment and plastic size increment. This results in the prediction model being able to reasonably describe the acceleration effect of crack propagation. In general, the prediction model of fatigue crack propagation proposed in this paper has relatively high accuracy with the increase in crack propagation length under different overload ratios.

## 6. Summary

The fatigue crack propagation behavior of thicker FMLs under typical overload was analyzed and predicted in this paper. By analyzing the characteristics of crack propagation behavior, the prediction models of the crack propagation rate under single-peak tensile/compressive overload were established, respectively.

The equivalent crack length model was improved to apply to thicker FMLs by considering the thickness size effect. The Wheeler model was modified to characterize the maximum stress in the Irwin model by introducing bridging stress. A prediction model of the crack propagation rate under single-peak tensile overload was established by combining the improved equivalent crack length model and the modified Wheeler model. Meanwhile, incremental plasticity theory was modified to describe the crack propagation behavior in the overload zone under compression overload in combination with the Wheeler model. A prediction model of the crack propagation rate under single-peak compressive overload was established by combining the improved equivalent crack length model and the modified incremental plasticity model. The overall prediction error rate of the proposed model under single-peak tensile/compressive overload is up to 9.7% and 8.1%, respectively.

In the actual physical damage process, crack propagation is accompanied by the changes in residual stress in the metal layer. The next step of this study will consider the time-varying nature of residual stress to further investigate the mechanism of crack propagation.

## Figures and Tables

**Table 1:** Chemical composition of 2060-T8 Al-Li alloy.

Chemical Composition	Li	Cu	Mg	Zn	Mn	Ag	Zr	Fe	Si	Al
Mass fraction	0.617%	3.469%	0.716%	0.326%	0.271%	0.249%	0.102%	0.026%	0.016%	the rest

**Table 2:** Properties of S4 fiber and SY-24 adhesive.

Material Category	Average Tensile Breaking Strength of the Yarn/MPa	Average Strength of the Impregnated Yarn/MPa	Content of Combustible Substances	Moisture Content	Fiber Volume Fraction	Average Lap Shear Strength/MPa	Average Peel Strength/KN/m
S4 fiber	≥780	≥3000	0.65~1.25%	≤0.2%	74.7%	-	-
SY-24 adhesive	-	-	-	-	-	≥30	≥6

**Table 3:** Mechanical properties of Al-Li alloy.

Material Category	Elastic Modulus E/GPa	Poisson’s Ratio	Tensile Strength $\sigma_{b}$/MPa	Yield Strength $\sigma_{s}$/MPa	Elongation at Break/%
Al-Li alloy	72.4	0.3	483	441	8
Prepreg layer	54.6	0.252	1735	-	-

## Data Availability

The raw data supporting the conclusions of this article will be made available by the authors on request.

## Nomenclature

FMLs	Fiber metal laminates
CA	Constant amplitude
TO	Tensile overload
CO	Compression overload
*R*_ol_	Overload ratio
*a*_ol_	Specified crack length
*S*_ol_	Corresponding loading
*S*_max_	Peak value of remote stress applied by the laminate
d*a*/d*N*	Crack propagation rate
*a*	Fatigue crack length
*N*	Loading cycle times
$a_{i}$	The crack propagation length at $N_{i}$ cycles
∆*K*_eff_	Effective stress intensity factor amplitude
*F*	Configuration factor of the laminate specimen
*s*	Length of sawing crack
*F*_0_	Value of *F* when the fatigue crack length at the sawing crack length
∆*S*	Remote stress amplitude of the laminate
*l*_0_	Equivalent crack length of the laminate
*C*_1_, *m*_1_, *n*_1_	Crack propagation constants for the component metals of the laminate
*R*_c_	Effective cyclic stress ratio for the component metals of the laminate
*S*_0_	Stress applied to the laminate when the actual stress of the component metals in FMLs is 0
*E*_la_	Elastic modulus of the laminate
*E*_Al_	Elastic modulus of the component metal
*σ*_r,Al_	Residual stress of the component metal
*k*	Thickness effect factor
*A*, *B*	Calculated from the crack propagation rate measured in the fatigue crack propagation test
${\left(\frac{da}{dN}\right)}_{\mathrm{wen}}$	Crack propagation rate for FMLs under constant amplitude loading
${\left(\frac{da}{dN}\right)}_{\mathrm{con}}$	Crack propagation rate for Fiber/Al-Li laminate under constant amplitude loading
${\left(\frac{da}{dN}\right)}_{\mathrm{ret}}$	Crack propagation rate in the crack propagation hysteresis period
*C*_p_	Crack propagation hysteresis coefficient
*m*	Crack hysteresis index
*R*_Y1_	Plastic zone generated by reference loading
*R*_Y2_	Plastic zone generated by tensile overload
$\rho_{\max}$	Maximum size of positive plastic zone at the crack tip
*K*_max_	Stress intensity factor under the maximum loading
*σ*_s_	Yield strength of metal materials
*σ*_ffective_	Effective stress in positive plastic zone
*σ*_max,com,Al_	Maximum compression loading of effective remote stress for the component metal
*σ*_max,com_	Maximum compression loading
*σ*_bridging_	Bridging stress
*σ*_remote_	Remote stress
*γ*	Material constant
$\rho_{r}$	Maximum size of reverse plastic zone at the crack tip
C_d_	Crack propagation acceleration coefficient
*h*	Crack acceleration index
